# Supplementary material for: Modeling glioblastoma heterogeneity as a dynamic network of cell states
Source: Mol Syst Biol. 2021 Sep 16;17(9):e10105. doi: 10.15252/msb.202010105 (PMC8444284; doi:10.15252/msb.202010105)
Supplement: Supplementary file 5 — Source Data for Figure 3 [file MSB-17-e10105-s001.zip › Figure3A_sourcedata/GSEA_3065/nosigdb_state3.GseaPreranked.1622718426028/NB_KI_CLUSTER8.html]

Details for gene set NB\_KI\_CLUSTER8[GSEA]

|  || Dataset | state3 |
| Phenotype | NoPhenotypeAvailable |
| Upregulated in class | na\_pos |
| GeneSet | NB\_KI\_CLUSTER8 |
| Enrichment Score (ES) | 0.4148412 |
| Normalized Enrichment Score (NES) | 1.859122 |
| Nominal p-value | 0.0 |
| FDR q-value | 0.0030144472 |
| FWER p-Value | 0.033 |
Table: GSEA Results Summary

  

Fig 1: Enrichment plot: NB\_KI\_CLUSTER8      
 Profile of the Running ES Score & Positions of GeneSet Members on the Rank Ordered List

  

| PROBE | GENE SYMBOL | GENE\_TITLE | RANK IN GENE LIST | RANK METRIC SCORE | RUNNING ES | CORE ENRICHMENT || 1 | SYNE2 |  |  | 16 | 0.529 | 0.0157 | Yes |
| 2 | HNRNPU |  |  | 71 | 0.373 | 0.0217 | Yes |
| 3 | MDM4 |  |  | 92 | 0.351 | 0.0310 | Yes |
| 4 | UBC |  |  | 145 | 0.313 | 0.0352 | Yes |
| 5 | MACF1 |  |  | 160 | 0.305 | 0.0437 | Yes |
| 6 | SUN1 |  |  | 174 | 0.297 | 0.0520 | Yes |
| 7 | SF3B1 |  |  | 180 | 0.295 | 0.0612 | Yes |
| 8 | DDX17 |  |  | 191 | 0.290 | 0.0696 | Yes |
| 9 | NKTR |  |  | 202 | 0.282 | 0.0778 | Yes |
| 10 | RDH11 |  |  | 212 | 0.277 | 0.0859 | Yes |
| 11 | SLC3A2 |  |  | 215 | 0.276 | 0.0949 | Yes |
| 12 | RBM6 |  |  | 216 | 0.275 | 0.1040 | Yes |
| 13 | TRIO |  |  | 235 | 0.269 | 0.1108 | Yes |
| 14 | PGAP1 |  |  | 259 | 0.260 | 0.1167 | Yes |
| 15 | HMGCS1 |  |  | 267 | 0.254 | 0.1243 | Yes |
| 16 | ATP2B1 |  |  | 271 | 0.253 | 0.1323 | Yes |
| 17 | ARID1B |  |  | 276 | 0.251 | 0.1402 | Yes |
| 18 | PHTF2 |  |  | 292 | 0.245 | 0.1466 | Yes |
| 19 | CCDC14 |  |  | 334 | 0.234 | 0.1495 | Yes |
| 20 | APMAP |  |  | 335 | 0.233 | 0.1572 | Yes |
| 21 | N4BP2L2 |  |  | 343 | 0.231 | 0.1641 | Yes |
| 22 | ZNF431 |  |  | 345 | 0.231 | 0.1716 | Yes |
| 23 | ZNF638 |  |  | 346 | 0.230 | 0.1792 | Yes |
| 24 | TMEM259 |  |  | 354 | 0.229 | 0.1860 | Yes |
| 25 | CD46 |  |  | 357 | 0.229 | 0.1934 | Yes |
| 26 | MFSD14C |  |  | 360 | 0.228 | 0.2007 | Yes |
| 27 | ZNF75A |  |  | 374 | 0.224 | 0.2066 | Yes |
| 28 | SPTAN1 |  |  | 376 | 0.223 | 0.2139 | Yes |
| 29 | TTC17 |  |  | 384 | 0.222 | 0.2204 | Yes |
| 30 | STIM2 |  |  | 427 | 0.213 | 0.2225 | Yes |
| 31 | WSB1 |  |  | 435 | 0.212 | 0.2287 | Yes |
| 32 | ELOVL5 |  |  | 450 | 0.209 | 0.2340 | Yes |
| 33 | POLR2A |  |  | 472 | 0.204 | 0.2383 | Yes |
| 34 | LENG8 |  |  | 485 | 0.201 | 0.2435 | Yes |
| 35 | KMT2A |  |  | 491 | 0.200 | 0.2496 | Yes |
| 36 | VPS13C |  |  | 493 | 0.200 | 0.2561 | Yes |
| 37 | REV3L |  |  | 506 | 0.198 | 0.2612 | Yes |
| 38 | TTC14 |  |  | 522 | 0.196 | 0.2660 | Yes |
| 39 | EPM2AIP1 |  |  | 531 | 0.194 | 0.2715 | Yes |
| 40 | KIAA1109 |  |  | 591 | 0.187 | 0.2707 | Yes |
| 41 | RANBP2 |  |  | 604 | 0.185 | 0.2754 | Yes |
| 42 | HERC1 |  |  | 636 | 0.181 | 0.2778 | Yes |
| 43 | PIGQ |  |  | 658 | 0.177 | 0.2812 | Yes |
| 44 | PCSK7 |  |  | 665 | 0.176 | 0.2863 | Yes |
| 45 | RBM5 |  |  | 672 | 0.175 | 0.2914 | Yes |
| 46 | CDK13 |  |  | 681 | 0.174 | 0.2962 | Yes |
| 47 | PCNX4 |  |  | 686 | 0.173 | 0.3015 | Yes |
| 48 | HCG18 |  |  | 689 | 0.173 | 0.3070 | Yes |
| 49 | OFD1 |  |  | 714 | 0.168 | 0.3097 | Yes |
| 50 | PRPF4B |  |  | 724 | 0.167 | 0.3142 | Yes |
| 51 | FRYL |  |  | 787 | 0.159 | 0.3122 | Yes |
| 52 | HNRNPH1 |  |  | 793 | 0.158 | 0.3168 | Yes |
| 53 | GIGYF1 |  |  | 830 | 0.155 | 0.3177 | Yes |
| 54 | CLK4 |  |  | 850 | 0.153 | 0.3205 | Yes |
| 55 | CCDC66 |  |  | 861 | 0.151 | 0.3244 | Yes |
| 56 | CPD |  |  | 868 | 0.151 | 0.3287 | Yes |
| 57 | VPS13A |  |  | 936 | 0.144 | 0.3255 | Yes |
| 58 | HSPH1 |  |  | 937 | 0.144 | 0.3303 | Yes |
| 59 | POGZ |  |  | 982 | 0.140 | 0.3297 | Yes |
| 60 | KDM5A |  |  | 984 | 0.139 | 0.3342 | Yes |
| 61 | CENPC |  |  | 992 | 0.139 | 0.3380 | Yes |
| 62 | SORL1 |  |  | 1004 | 0.138 | 0.3413 | Yes |
| 63 | PHC3 |  |  | 1017 | 0.137 | 0.3444 | Yes |
| 64 | MPHOSPH9 |  |  | 1019 | 0.137 | 0.3489 | Yes |
| 65 | TMEM245 |  |  | 1072 | 0.133 | 0.3471 | Yes |
| 66 | GPR155 |  |  | 1095 | 0.130 | 0.3488 | Yes |
| 67 | YTHDC2 |  |  | 1121 | 0.128 | 0.3501 | Yes |
| 68 | CDC42BPA |  |  | 1141 | 0.127 | 0.3521 | Yes |
| 69 | INSR |  |  | 1144 | 0.126 | 0.3561 | Yes |
| 70 | SNRNP70 |  |  | 1148 | 0.126 | 0.3599 | Yes |
| 71 | UBR5 |  |  | 1173 | 0.124 | 0.3612 | Yes |
| 72 | ZNF675 |  |  | 1217 | 0.120 | 0.3601 | Yes |
| 73 | GLMN |  |  | 1241 | 0.119 | 0.3613 | Yes |
| 74 | SRRT |  |  | 1256 | 0.118 | 0.3635 | Yes |
| 75 | OGA |  |  | 1266 | 0.117 | 0.3663 | Yes |
| 76 | PABPN1 |  |  | 1273 | 0.117 | 0.3695 | Yes |
| 77 | ANKRD36 |  |  | 1314 | 0.114 | 0.3686 | Yes |
| 78 | TNRC6B |  |  | 1326 | 0.114 | 0.3710 | Yes |
| 79 | EXOSC10 |  |  | 1355 | 0.111 | 0.3714 | Yes |
| 80 | SETD2 |  |  | 1362 | 0.111 | 0.3744 | Yes |
| 81 | MAT2A |  |  | 1386 | 0.109 | 0.3753 | Yes |
| 82 | AAK1 |  |  | 1408 | 0.108 | 0.3763 | Yes |
| 83 | ZNF430 |  |  | 1435 | 0.106 | 0.3768 | Yes |
| 84 | ODF2L |  |  | 1454 | 0.105 | 0.3782 | Yes |
| 85 | GTF2H2 |  |  | 1458 | 0.105 | 0.3813 | Yes |
| 86 | SHPRH |  |  | 1505 | 0.103 | 0.3793 | Yes |
| 87 | LTN1 |  |  | 1529 | 0.101 | 0.3799 | Yes |
| 88 | RNF19A |  |  | 1530 | 0.101 | 0.3833 | Yes |
| 89 | BIRC6 |  |  | 1534 | 0.101 | 0.3863 | Yes |
| 90 | FTX |  |  | 1541 | 0.101 | 0.3889 | Yes |
| 91 | UBR2 |  |  | 1549 | 0.101 | 0.3914 | Yes |
| 92 | XRN1 |  |  | 1586 | 0.098 | 0.3904 | Yes |
| 93 | ATF7IP |  |  | 1612 | 0.097 | 0.3907 | Yes |
| 94 | RALGAPA1 |  |  | 1620 | 0.096 | 0.3930 | Yes |
| 95 | DNAJB14 |  |  | 1626 | 0.096 | 0.3956 | Yes |
| 96 | BROX |  |  | 1660 | 0.094 | 0.3948 | Yes |
| 97 | QTRT1 |  |  | 1661 | 0.093 | 0.3979 | Yes |
| 98 | SLC35A3 |  |  | 1684 | 0.093 | 0.3984 | Yes |
| 99 | ZC3H7A |  |  | 1706 | 0.091 | 0.3990 | Yes |
| 100 | RICTOR |  |  | 1760 | 0.088 | 0.3956 | Yes |
| 101 | RABL2B |  |  | 1763 | 0.088 | 0.3983 | Yes |
| 102 | BTAF1 |  |  | 1771 | 0.088 | 0.4004 | Yes |
| 103 | CDS2 |  |  | 1794 | 0.087 | 0.4007 | Yes |
| 104 | UBE2G2 |  |  | 1826 | 0.085 | 0.3998 | Yes |
| 105 | ZNF7 |  |  | 1827 | 0.085 | 0.4027 | Yes |
| 106 | MED17 |  |  | 1841 | 0.084 | 0.4039 | Yes |
| 107 | TRA2A |  |  | 1849 | 0.084 | 0.4059 | Yes |
| 108 | CDK10 |  |  | 1851 | 0.084 | 0.4085 | Yes |
| 109 | TRMT13 |  |  | 1852 | 0.084 | 0.4113 | Yes |
| 110 | CDK11B |  |  | 1900 | 0.081 | 0.4084 | Yes |
| 111 | MKLN1 |  |  | 1902 | 0.081 | 0.4110 | Yes |
| 112 | PRPF3 |  |  | 1935 | 0.079 | 0.4098 | Yes |
| 113 | GCC2 |  |  | 1944 | 0.078 | 0.4115 | Yes |
| 114 | PPP4R3A |  |  | 1971 | 0.077 | 0.4110 | Yes |
| 115 | ELMSAN1 |  |  | 2004 | 0.076 | 0.4097 | Yes |
| 116 | BRAF |  |  | 2012 | 0.076 | 0.4114 | Yes |
| 117 | PHACTR4 |  |  | 2021 | 0.075 | 0.4129 | Yes |
| 118 | SRSF6 |  |  | 2027 | 0.075 | 0.4148 | Yes |
| 119 | MPDZ |  |  | 2069 | 0.073 | 0.4124 | Yes |
| 120 | TSNAX |  |  | 2090 | 0.072 | 0.4124 | Yes |
| 121 | ZNF791 |  |  | 2091 | 0.072 | 0.4148 | Yes |
| 122 | KAT6A |  |  | 2148 | 0.070 | 0.4105 | No |
| 123 | TOM1L2 |  |  | 2160 | 0.070 | 0.4116 | No |
| 124 | FBXO42 |  |  | 2194 | 0.069 | 0.4099 | No |
| 125 | CHD2 |  |  | 2197 | 0.069 | 0.4120 | No |
| 126 | GPATCH2L |  |  | 2216 | 0.068 | 0.4121 | No |
| 127 | RNF145 |  |  | 2234 | 0.067 | 0.4123 | No |
| 128 | CHORDC1 |  |  | 2288 | 0.065 | 0.4082 | No |
| 129 | LMBRD1 |  |  | 2291 | 0.065 | 0.4101 | No |
| 130 | TRIB2 |  |  | 2296 | 0.065 | 0.4118 | No |
| 131 | PPP1R10 |  |  | 2331 | 0.063 | 0.4098 | No |
| 132 | PRPF38B |  |  | 2336 | 0.063 | 0.4115 | No |
| 133 | CDK12 |  |  | 2343 | 0.063 | 0.4128 | No |
| 134 | ADNP |  |  | 2362 | 0.062 | 0.4128 | No |
| 135 | IFRD1 |  |  | 2444 | 0.059 | 0.4052 | No |
| 136 | LAS1L |  |  | 2472 | 0.058 | 0.4039 | No |
| 137 | FAM131A |  |  | 2489 | 0.057 | 0.4039 | No |
| 138 | FAM53C |  |  | 2503 | 0.057 | 0.4042 | No |
| 139 | ICE2 |  |  | 2525 | 0.056 | 0.4036 | No |
| 140 | BTG3 |  |  | 2610 | 0.053 | 0.3954 | No |
| 141 | XRRA1 |  |  | 2626 | 0.052 | 0.3954 | No |
| 142 | DDHD1 |  |  | 2634 | 0.052 | 0.3963 | No |
| 143 | RBM14 |  |  | 2672 | 0.051 | 0.3936 | No |
| 144 | PTPN4 |  |  | 2685 | 0.050 | 0.3939 | No |
| 145 | WEE1 |  |  | 2687 | 0.050 | 0.3954 | No |
| 146 | PILRB |  |  | 2689 | 0.050 | 0.3970 | No |
| 147 | MLLT6 |  |  | 2706 | 0.050 | 0.3967 | No |
| 148 | DPF2 |  |  | 2726 | 0.049 | 0.3961 | No |
| 149 | GAN |  |  | 2727 | 0.049 | 0.3978 | No |
| 150 | SACM1L |  |  | 2739 | 0.049 | 0.3981 | No |
| 151 | NPAT |  |  | 2758 | 0.048 | 0.3976 | No |
| 152 | ZMYM2 |  |  | 2782 | 0.048 | 0.3964 | No |
| 153 | ABCA5 |  |  | 2815 | 0.046 | 0.3942 | No |
| 154 | RNF168 |  |  | 2857 | 0.045 | 0.3908 | No |
| 155 | NFATC3 |  |  | 2860 | 0.045 | 0.3921 | No |
| 156 | FAR1 |  |  | 2862 | 0.045 | 0.3935 | No |
| 157 | KIAA0355 |  |  | 2894 | 0.044 | 0.3913 | No |
| 158 | RBPJ |  |  | 2897 | 0.044 | 0.3925 | No |
| 159 | PPP4R1 |  |  | 2907 | 0.044 | 0.3929 | No |
| 160 | MSL1 |  |  | 2916 | 0.044 | 0.3934 | No |
| 161 | ANKRD26 |  |  | 2918 | 0.043 | 0.3947 | No |
| 162 | MIER1 |  |  | 2919 | 0.043 | 0.3962 | No |
| 163 | HERPUD2 |  |  | 2978 | 0.041 | 0.3907 | No |
| 164 | STX16 |  |  | 3016 | 0.041 | 0.3877 | No |
| 165 | PRDM2 |  |  | 3027 | 0.040 | 0.3878 | No |
| 166 | CEP85L |  |  | 3066 | 0.039 | 0.3846 | No |
| 167 | PHF20L1 |  |  | 3135 | 0.038 | 0.3778 | No |
| 168 | CYHR1 |  |  | 3176 | 0.036 | 0.3743 | No |
| 169 | PIAS1 |  |  | 3211 | 0.035 | 0.3715 | No |
| 170 | DIP2A |  |  | 3218 | 0.035 | 0.3719 | No |
| 171 | NUMA1 |  |  | 3232 | 0.035 | 0.3716 | No |
| 172 | IVNS1ABP |  |  | 3239 | 0.035 | 0.3720 | No |
| 173 | MYO9A |  |  | 3247 | 0.035 | 0.3723 | No |
| 174 | RABGAP1 |  |  | 3272 | 0.034 | 0.3706 | No |
| 175 | SRSF3 |  |  | 3283 | 0.034 | 0.3705 | No |
| 176 | NEMF |  |  | 3360 | 0.032 | 0.3626 | No |
| 177 | PRKD3 |  |  | 3436 | 0.030 | 0.3547 | No |
| 178 | CWF19L2 |  |  | 3442 | 0.029 | 0.3551 | No |
| 179 | EIF4A3 |  |  | 3479 | 0.029 | 0.3518 | No |
| 180 | ATXN1 |  |  | 3504 | 0.028 | 0.3499 | No |
| 181 | CNOT9 |  |  | 3541 | 0.027 | 0.3465 | No |
| 182 | KPNA1 |  |  | 3586 | 0.026 | 0.3422 | No |
| 183 | PIK3R1 |  |  | 3604 | 0.025 | 0.3410 | No |
| 184 | UBR1 |  |  | 3634 | 0.025 | 0.3384 | No |
| 185 | TFDP2 |  |  | 3647 | 0.024 | 0.3378 | No |
| 186 | HTT |  |  | 3695 | 0.023 | 0.3330 | No |
| 187 | TLE3 |  |  | 3709 | 0.022 | 0.3322 | No |
| 188 | ARHGAP5 |  |  | 3722 | 0.022 | 0.3315 | No |
| 189 | ZBTB44 |  |  | 3728 | 0.022 | 0.3316 | No |
| 190 | PUM2 |  |  | 3746 | 0.021 | 0.3303 | No |
| 191 | SNRK |  |  | 3792 | 0.020 | 0.3257 | No |
| 192 | ANKZF1 |  |  | 3816 | 0.020 | 0.3236 | No |
| 193 | OGFRL1 |  |  | 3920 | 0.017 | 0.3120 | No |
| 194 | EPS15 |  |  | 3980 | 0.016 | 0.3055 | No |
| 195 | CCNG2 |  |  | 4001 | 0.015 | 0.3036 | No |
| 196 | SRSF11 |  |  | 4003 | 0.015 | 0.3040 | No |
| 197 | BTBD7 |  |  | 4044 | 0.014 | 0.2998 | No |
| 198 | JADE1 |  |  | 4085 | 0.013 | 0.2955 | No |
| 199 | GNL3 |  |  | 4103 | 0.012 | 0.2939 | No |
| 200 | WNK1 |  |  | 4143 | 0.011 | 0.2896 | No |
| 201 | DXO |  |  | 4222 | 0.009 | 0.2807 | No |
| 202 | DGKZ |  |  | 4225 | 0.009 | 0.2807 | No |
| 203 | PHF1 |  |  | 4241 | 0.008 | 0.2792 | No |
| 204 | KLF12 |  |  | 4276 | 0.007 | 0.2755 | No |
| 205 | CALCOCO1 |  |  | 4294 | 0.007 | 0.2737 | No |
| 206 | AP3M2 |  |  | 4300 | 0.007 | 0.2733 | No |
| 207 | CCAR2 |  |  | 4312 | 0.006 | 0.2722 | No |
| 208 | INO80D |  |  | 4314 | 0.006 | 0.2723 | No |
| 209 | PIK3C2A |  |  | 4362 | 0.005 | 0.2669 | No |
| 210 | CLASP1 |  |  | 4435 | 0.003 | 0.2585 | No |
| 211 | CBX6 |  |  | 4460 | 0.002 | 0.2557 | No |
| 212 | PDE9A |  |  | 4471 | 0.002 | 0.2546 | No |
| 213 | MECP2 |  |  | 4485 | 0.002 | 0.2531 | No |
| 214 | ZBTB10 |  |  | 4501 | 0.001 | 0.2514 | No |
| 215 | SENP5 |  |  | 4502 | 0.001 | 0.2514 | No |
| 216 | RBBP6 |  |  | 4505 | 0.001 | 0.2513 | No |
| 217 | SEC22B |  |  | 4515 | 0.001 | 0.2502 | No |
| 218 | USP10 |  |  | 4523 | 0.001 | 0.2494 | No |
| 219 | PRKX |  |  | 4561 | -0.000 | 0.2451 | No |
| 220 | CREBRF |  |  | 4622 | -0.002 | 0.2380 | No |
| 221 | KIAA2026 |  |  | 4701 | -0.004 | 0.2289 | No |
| 222 | PURB |  |  | 4709 | -0.004 | 0.2282 | No |
| 223 | MEF2A |  |  | 4745 | -0.005 | 0.2242 | No |
| 224 | TAF3 |  |  | 4760 | -0.005 | 0.2227 | No |
| 225 | ZNF184 |  |  | 4776 | -0.005 | 0.2211 | No |
| 226 | NCSTN |  |  | 4795 | -0.006 | 0.2192 | No |
| 227 | TRIM8 |  |  | 4812 | -0.006 | 0.2175 | No |
| 228 | IRF2BP2 |  |  | 4927 | -0.009 | 0.2043 | No |
| 229 | LATS1 |  |  | 4964 | -0.010 | 0.2004 | No |
| 230 | PPM1K |  |  | 5046 | -0.012 | 0.1912 | No |
| 231 | CLASRP |  |  | 5123 | -0.014 | 0.1827 | No |
| 232 | SCRN2 |  |  | 5270 | -0.018 | 0.1660 | No |
| 233 | SEC22C |  |  | 5339 | -0.019 | 0.1586 | No |
| 234 | PRMT2 |  |  | 5354 | -0.020 | 0.1576 | No |
| 235 | LYSMD2 |  |  | 5367 | -0.020 | 0.1568 | No |
| 236 | RANBP9 |  |  | 5385 | -0.021 | 0.1555 | No |
| 237 | STRN3 |  |  | 5471 | -0.023 | 0.1462 | No |
| 238 | CAPN7 |  |  | 5504 | -0.024 | 0.1433 | No |
| 239 | TTC39C |  |  | 5543 | -0.026 | 0.1396 | No |
| 240 | GNB5 |  |  | 5561 | -0.026 | 0.1385 | No |
| 241 | SATB1 |  |  | 5608 | -0.027 | 0.1339 | No |
| 242 | FAM107B |  |  | 5624 | -0.028 | 0.1331 | No |
| 243 | SLBP |  |  | 5661 | -0.029 | 0.1298 | No |
| 244 | PURA |  |  | 5674 | -0.029 | 0.1293 | No |
| 245 | TBCC |  |  | 5748 | -0.031 | 0.1217 | No |
| 246 | SNN |  |  | 5791 | -0.032 | 0.1178 | No |
| 247 | SEPT9 |  |  | 5792 | -0.033 | 0.1189 | No |
| 248 | IRF2BPL |  |  | 5794 | -0.033 | 0.1199 | No |
| 249 | TFCP2 |  |  | 5813 | -0.033 | 0.1188 | No |
| 250 | MAX |  |  | 5913 | -0.036 | 0.1083 | No |
| 251 | CDKN2AIP |  |  | 5921 | -0.036 | 0.1087 | No |
| 252 | TRIM69 |  |  | 5949 | -0.037 | 0.1068 | No |
| 253 | GABARAPL1 |  |  | 5976 | -0.039 | 0.1050 | No |
| 254 | ZNF639 |  |  | 5998 | -0.040 | 0.1038 | No |
| 255 | RPA1 |  |  | 6115 | -0.044 | 0.0915 | No |
| 256 | MLLT3 |  |  | 6127 | -0.044 | 0.0917 | No |
| 257 | CPNE1 |  |  | 6174 | -0.045 | 0.0877 | No |
| 258 | PAK1 |  |  | 6189 | -0.046 | 0.0876 | No |
| 259 | BCAS2 |  |  | 6217 | -0.047 | 0.0860 | No |
| 260 | USE1 |  |  | 6290 | -0.049 | 0.0791 | No |
| 261 | H2AFX |  |  | 6437 | -0.054 | 0.0636 | No |
| 262 | TRA2B |  |  | 6483 | -0.056 | 0.0601 | No |
| 263 | PSMA1 |  |  | 6529 | -0.057 | 0.0567 | No |
| 264 | MRFAP1L1 |  |  | 6564 | -0.059 | 0.0546 | No |
| 265 | SEPT6 |  |  | 6642 | -0.063 | 0.0476 | No |
| 266 | CYB5B |  |  | 6751 | -0.068 | 0.0371 | No |
| 267 | MKNK2 |  |  | 6833 | -0.072 | 0.0299 | No |
| 268 | YME1L1 |  |  | 6862 | -0.074 | 0.0291 | No |
| 269 | FYN |  |  | 6924 | -0.077 | 0.0244 | No |
| 270 | MAPK1 |  |  | 6941 | -0.078 | 0.0251 | No |
| 271 | IDS |  |  | 6974 | -0.079 | 0.0239 | No |
| 272 | SNX10 |  |  | 6975 | -0.079 | 0.0266 | No |
| 273 | SMIM27 |  |  | 6984 | -0.079 | 0.0283 | No |
| 274 | TANK |  |  | 6995 | -0.080 | 0.0297 | No |
| 275 | NOSIP |  |  | 7063 | -0.085 | 0.0246 | No |
| 276 | DCTN6 |  |  | 7073 | -0.085 | 0.0264 | No |
| 277 | YES1 |  |  | 7119 | -0.088 | 0.0240 | No |
| 278 | CCDC59 |  |  | 7197 | -0.093 | 0.0180 | No |
| 279 | MINDY2 |  |  | 7310 | -0.101 | 0.0081 | No |
| 280 | ARRDC3 |  |  | 7386 | -0.107 | 0.0028 | No |
| 281 | CITED2 |  |  | 7430 | -0.111 | 0.0013 | No |
| 282 | PKN1 |  |  | 7567 | -0.124 | -0.0106 | No |
| 283 | EVL |  |  | 7641 | -0.132 | -0.0149 | No |
| 284 | C6orf62 |  |  | 7698 | -0.139 | -0.0169 | No |
| 285 | C9orf78 |  |  | 7728 | -0.143 | -0.0156 | No |
| 286 | SEPT1 |  |  | 7745 | -0.145 | -0.0127 | No |
| 287 | ARL4A |  |  | 7829 | -0.155 | -0.0173 | No |
| 288 | HSPE1 |  |  | 7918 | -0.170 | -0.0221 | No |
| 289 | ABHD17A |  |  | 7940 | -0.175 | -0.0188 | No |
| 290 | TPM3 |  |  | 7943 | -0.175 | -0.0132 | No |
| 291 | MXD4 |  |  | 7990 | -0.183 | -0.0126 | No |
| 292 | WTAP |  |  | 8000 | -0.186 | -0.0075 | No |
| 293 | STX10 |  |  | 8007 | -0.187 | -0.0020 | No |
| 294 | NETO2 |  |  | 8118 | -0.208 | -0.0081 | No |
| 295 | MAZ |  |  | 8350 | -0.273 | -0.0264 | No |
| 296 | MRPS6 |  |  | 8413 | -0.300 | -0.0238 | No |
| 297 | CDC42EP3 |  |  | 8473 | -0.326 | -0.0199 | No |
| 298 | SOD1 |  |  | 8526 | -0.349 | -0.0145 | No |
| 299 | C12orf75 |  |  | 8548 | -0.363 | -0.0049 | No |
| 300 | AES |  |  | 8597 | -0.398 | 0.0026 | No |
| 301 | ARL4C |  |  | 8660 | -0.467 | 0.0108 | No |
Table: GSEA details [plain text format]

  

Fig 2: NB\_KI\_CLUSTER8: Random ES distribution      
 Gene set null distribution of ES for **NB\_KI\_CLUSTER8**
